# Value of Serum NEUROG1 Methylation for the Detection of Advanced Adenomas and Colorectal Cancer

**DOI:** 10.3390/diagnostics10070437

**Published:** 2020-06-28

**Authors:** Olalla Otero-Estévez, María Gallardo-Gomez, María Páez de la Cadena, Francisco Javier Rodríguez-Berrocal, Joaquín Cubiella, Vicent Hernandez Ramirez, Laura García-Nimo, Loretta De Chiara

**Affiliations:** 1 Department of Biochemistry, Genetics and Immunology, University of Vigo, 36310 Vigo, Spain; olalla.otero@uvigo.es (O.O.-E.); mgallardo@uvigo.es (M.G.-G.); mpaez@uvigo.es (M.P.d.l.C.); berrocal@uvigo.es (F.J.R.-B.); 2 Centro de Investigaciones Biomédicas, Centro Singular de Investigación de Galicia, University of Vigo, 36310 Vigo, Spain; 3 Department of Gastroenterology, Complexo Hospitalario Universitario de Ourense, Instituto de Investigación Biomédica Galicia Sur, Centro de Investigación Biomédica en Red de Enfermedades Hepáticas y Digestivas (CIBERehd), 32005 Ourense, Spain; joaquin.cubiella.fernandez@sergas.es; 4 Department of Gastroenterology, Xerencia de Xestión Integrada de Vigo, Instituto de Investigación Biomédica Galicia Sur, 36213 Vigo, Spain; vicent.hernandez.ramirez@sergas.es; 5 Department of Clinical Analysis, Complexo Hospitalario Universitario de Ourense, Instituto de Investigación Biomédica Galicia Sur, 32005 Ourense, Spain; laura.garcia.nimo@sergas.es

**Keywords:** DNA methylation, NEUROG1, colorectal cancer, advanced adenomas, screening, serum biomarker, FIT

## Abstract

Aberrant DNA methylation detected in liquid biopsies is a promising approach for colorectal cancer (CRC) detection, including premalignant advanced adenomas (AA). We evaluated the diagnostic capability of serum NEUROG1 methylation for the detection of AA and CRC. A CpG island in NEUROG1 promoter was assessed by bisulfite pyrosequencing in a case-control cohort to select optimal CpGs. Selected sites were evaluated through a nested methylation-specific qPCR custom assay in a screening cohort of 504 asymptomatic family-risk individuals. Individuals with no colorectal findings and benign pathologies showed low serum NEUROG1 methylation, similar to non-advanced adenomas. Contrarily, individuals bearing AA or CRC (advanced neoplasia—AN), exhibited increased NEUROG1 methylation. Using >1.3518% as NEUROG1 cut-off (90.60% specificity), 33.33% of AN and 32.08% of AA were identified, detecting 50% CRC cases. Nonetheless, the combination of NEUROG1 with fecal immunochemical test (FIT), together with age and gender through a multivariate logistic regression resulted in an AUC = 0.810 for AN, and 0.796 for AA, detecting all cancer cases and 35–47% AA (specificity 98–95%). The combination of NEUROG1 methylation with FIT, age and gender demonstrated a convenient performance for the detection of CRC and AA, providing a valuable tool for CRC screening programs in asymptomatic individuals.

## 1. Introduction

Colorectal cancer (CRC) is one of the most common cancers worldwide, accounting for over one million new cases each year [1]. Neoplastic transformation from precancerous adenomas to cancer can last decades, providing the opportunity to implement screening strategies that could reduce CRC incidence and mortality [2,3]. Screening is especially important in individuals with a family history of CRC because of the increased risk of developing CRC and adenomas [4,5]. The most used non-invasive screening test is the fecal immunochemical test (FIT), despite its limited sensitivity for premalignant advanced adenomas (AA) mainly due to the intermittent and infrequent bleeding of these lesions [6,7,8]. Moreover, as bleeding from the lower intestinal tract is a symptom related to conditions like diverticular disease, colitis, Crohn′s disease and anorectal disorders, false-positive results may be increased [9]. Another limitation of FIT is its dependence on localization, resulting more useful for distal lesions compared with proximal ones [10]. Therefore, there is an imperative need for the identification of non-invasive, blood-based markers that can help in the detection of cancer and AA.

The presence of widespread CpG island methylation in a tumor, known as CpG island methylator phenotype (CIMP), has been described in colorectal cancer biology, which is useful for the understanding of carcinogenic pathways [11,12]. Several CIMP gene panels have been proposed, some including NEUROG1 as one of the frequently methylated genes [13,14,15]. The NEUROG1 gene, located on chromosome 5 (5q23–q31), encodes for a transcriptional factor that binds to E box elements. Methylation analysis of NEUROG1 in CRC tumors showed progressive hypermethylation associated with neoplastic development, from normal mucosa–hyperplastic polyp–adenoma–primary carcinoma, and finally metastatic colorectal carcinoma, showing the highest methylation [16].

Aberrant DNA methylation has been described in liquid biopsies, resulting in a feasible approach to provide new potential biomarkers for CRC screening [17,18]. In a small cohort Herbst et al. [19] found that methylation of NEUROG1 in serum could discriminate healthy individuals from CRC patients, suggesting its utility for CRC diagnosis. However, no further reports have confirmed these findings in a large population, nor have extended the study to the diagnosis of premalignant lesions.

The aim of this study was to evaluate serum NEUROG1 methylation and analyze the diagnostic capability for the detection of AA and CRC in a cohort of asymptomatic individuals with at least one first-degree relative (FDR) with CRC. In our study we demonstrate that serum NEUROG1 methylation could be useful for the detection of AA and cancer. A convenient performance was found when NEUROG1 was combined with data from FIT, age and gender, providing a valuable tool for CRC screening programs in asymptomatic individuals.

## 2. Materials and Methods

### 2.1. Study Population and Study Design

**Figure 1:**
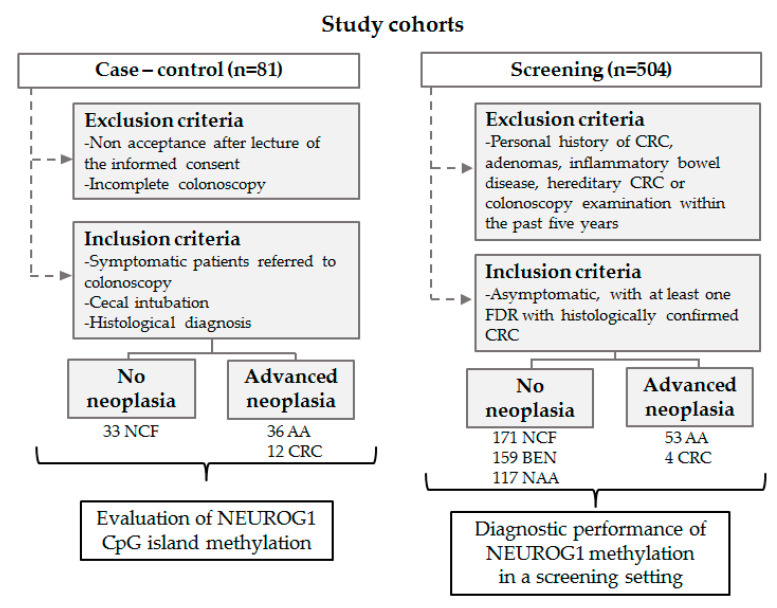
Study design flow-chart. FDR: first-degree relative; NCF: no colorectal findings; BEN: benign pathology; NAA: non-advanced adenomas; AA: advanced adenomas; CRC: colorectal cancer.

The study included two different cohorts: A case-control and a screening cohort. The case-control cohort was used to evaluate individual CpG sites in NEUROG1 promoter using bisulfite pyrosequencing and optimize the CpG sites with increased discriminatory capability, while the screening cohort was used to evaluate and validate the selected CpG sites through a nested methylation-specific qPCR. The study design is summarized in Figure 1, as well as the exclusion and inclusion criteria.

The case-control cohort included 12 symptomatic CRC cases (4 stage I, 3 stage II and 5 stage III, classified according to the AJCC staging system [20], 36 individuals with AA and 33 individuals with no colorectal findings from Complexo Hospitalario Universitario de Ourense. Controls (individuals with no colorectal findings) included 16 men and 17 women (median age 54.25 years), while CRC and AA cases included 29 men and 19 women (median age 58.19 years).

The screening cohort included 504 asymptomatic individuals with at least one FDR with confirmed CRC (205 men and 299 females, median age 54.45 years).

Individuals from this prospective, controlled, double-blinded study were also recruited from Complexo Hospitalario Universitario de Ourense and were referred to each undertake a colonoscopy and a FIT, besides a blood extraction to obtain serum. Following colonoscopy, individuals were classified as: 171 with no colorectal findings, 159 with benign pathologies (4 inflammatory polyps, 38 hyperplastic polyps, 65 hemorrhoids, 46 diverticula, and 6 with other benign pathologies), 117 with non-advanced adenomas, 53 AA and 4 CRC cases (two stage I, one stage II, and one stage III). AA included adenomas ≥10 mm, with villous component or high-grade dysplasia. CRC and AA cases were referred as advanced neoplasia (AN). Lesions were classified as ‘proximal’ when located only proximal to the splenic flexure, and ‘distal’ when found only in the distal or in both the distal and proximal colon.

The study followed the clinical and ethical practices of the Spanish Government and the Helsinki Declaration, and was approved by the Galician Ethical Committee for Clinical Research. Informed consent was obtained from each individual and anonymity was warranted.

### 2.2. Blood Samples and Stool Samples

Blood and a stool sample were obtained one week before colonoscopy. Blood samples were coagulated at room temperature for 20 min, and centrifuged at 2000× *g* for 15 min. Serum was stored at −20 °C. There were no diet or medication restrictions for stool collection.

The fecal occult blood (µg hemoglobin/g feces) was measured using a quantitative immunological test for the automated OC-Sensor (Eiken Chemical, Tokyo, Japan).

### 2.3. DNA Extraction and Sodium Bisulfite Modification

Genomic DNA was isolated from 300–1000 μL serum using QIAamp DNA Blood Mini Kit (Qiagen, Hilden, Germany) and was bisulfite converted using EZ DNA Methylation-Direct kit (Zymo Research, Irvine, CA, USA), following the manufacturers′ protocol. Modified DNA was stored at −80 °C.

A fully-methylated control was prepared from DNA extracted from peripheral blood obtained from a control individual, and treated with CpG methyltransferase (M.SssI; New England Biolabs, Ipswich, MA, USA). An unmethylated control using the same DNA, not treated with M.SssI, was also prepared. Both fully-methylated and unmethylated controls were treated with sodium bisulfite, and their methylation status, at least for the region analyzed in the NEUROG1 promoter, was corroborated through bisulfite pyrosequencing.

### 2.4. Bisulfite Pyrosequencing

**Figure 2:**
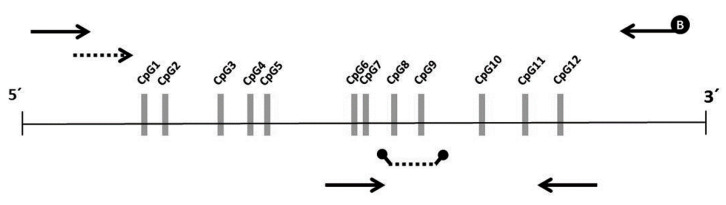
Representation of the CpG island analyzed located in the promoter of NEUROG1. The relative position of the 12 CpG sites is shown. Forward and reverse primers (solid arrows), and sequencing primer (dashed line) used for bisulphite pyrosequencing (B = biotin) is shown on the top of the figure. Forward and reverse primers (solid arrows), and probe (dashed line) used for the MS-qPCR is shown on the bottom of the figure.

Bisulfite pyrosequencing was performed for quantitative methylation analysis of the CpG island (including 12 CpG sites—Figure 2) in the NEUROG1 promoter, previously analyzed by others [13,19]. Methylation differences at each single CpG site was evaluated between individuals with no colorectal pathology and AN. The combination of CpG sites that better discriminated these groups was selected.

PCR primers externally targeting the region of interest (191 bp), besides the sequencing primer, were designed using Pyromark Assay Design software v2.0 (Qiagen, Hilden, Germany). In each amplification set, a fully-methylated control, an unmethylated control and a no template control were included, besides the samples from the case-control cohort. PCR condition and primer sequences are described in the Supplementary Materials.

The sequencing reaction and quantification of methylation were conducted using the PyroMark MD instrument, and analyzed with the Pyro Q-CpG software (Qiagen, Hilden, Germany). A methylation percentage was obtained for each CpG site interrogated, per sample.

### 2.5. Nested Methylation-Specific qPCR

NEUROG1 methylation was quantified at the CpG sites selected by bisulfite pyrosequencing, using a custom qPCR approach. The first pre-amplification step targeted the region of interest, followed by a MS-qPCR (methylation-specific qPCR), using diluted pre-amplification products as template. Further details are provided in the Supplementary Materials.

A standard curve for NEUROG1 methylation was elaborated with dilutions of the fully-methylated control (100−0.1% methylation; amplification efficiency = 96.16%; slope = −3.418; R^2^ = 0.9996). A linear fit of the mean Cq (quantification cycle) as a function of the log10 methylation percentage was obtained, and a non-normalized methylation percentage was estimated for each sample. To normalize for input DNA across the samples, the ACTB gene was used in a nested qPCR, and a relative DNA quantity was obtained (Supplementary Material).

The normalized methylation percentage (NMP) for NEUROG1 was calculated using this formula:

(1) $$NMP=\frac{Non-normalized\;methylation\;percentage}{Relative\;DNA\;quantity}\times 100$$

### 2.6. Statistical Analysis

Mean, standard deviation, median and interquartile range (IQR) were presented for continuous variables. Non-parametric statistic was used for two-sample group comparisons (Mann–Whitney U test).

Receiver–operating characteristic (ROC) curves were used to evaluate the ability of the marker to discriminate AN or AA from individuals with no neoplasia, providing the area under the curve (AUC). The cut-offs selected resulted from setting specificity close to 90%, 95% or 98%, prioritizing specificity instead of sensitivity. This criterion guaranteed a low proportion of false positives, which is highly desirable for a colorectal cancer screening test. Sensitivity, and negative and positive predictive values were estimated for the fixed specificity values. Logistic regression models based on NEUROG1 and/or FIT transformed to log10 (marker + 2), were elaborated, including gender and age as confounders. Differences in model fit were evaluated with a likelihood ratio test. AUC values were compared using the method of DeLong. McNemar test was used to compare the proportion of AN or AA cases detected, while Fisher′s exact test was employed to compare the proportion of distal and proximal lesions detected. Statistical analyses were done with the SPSS software (v.20.0; Chicago, IL, USA), R environment (v.3.6.3) and MedCalc Software (v.14.12.0; Oostende, Belgium). All tests were two-sided and *p*-values ≤ 0.05 were considered statistically significant.

## 3. Results

### 3.1. Selection of CpG Sites in Serum NEUROG1 in the Case-Control Cohort

The methylation status of NEUROG1, based on bisulfite pyrosequencing, was analyzed to evaluate the individual discriminatory capability of each of the 12 CpG sites interrogated. Methylation values were skewed towards 0% and did not follow a normal distribution. Methylation differences at each single CpG site was evaluated in individuals with no colorectal pathology and AN. Mean and median methylation for each CpG site are shown in Supplementary Table S1. All CpG sites showed increased methylation in the AN group compared to controls, with statistically significant differences only for CpG 9. The highest AUC values for the detection of AN were found for CpG 7, 8, 9 and 12, ranging from 0.573 to 0.633. Hence, the mean methylation percentage of these four sites resulted in significant differences when comparing no colorectal findings and AN, with an AUC value of 0.652 (Supplementary Table S1). Based on this result, the methylation analysis of NEUROG1 using MS-qPCR was restricted to CpG sites 7, 8, 9 and 12.

### 3.2. Methylation Analysis of Serum NEUROG1 in the Screening Cohort

Methylation analysis by MS-qPCR of the selected CpG sites, quantified in 504 individuals, did not show a normal distribution, resulting in 73.02% of the cases with 0% methylation. Methylation levels analyzed according to demographic variables (Supplementary Table S2) indicated that older individuals and males showed slightly higher NEUROG1 methylation, though no statistically significant. No differences regarding familial risk were found.

In relation to the colorectal findings groups (Table 1), 0.00% (0.00–0.00%) median and IQR methylation of NEUROG1 resulted in individuals with no colorectal findings and all the benign pathology sub-groups (inflammatory and hyperplastic polyps, hemorrhoids, diverticula and other benign pathologies). When methylation was compared between groups with no colorectal findings and benign sub-groups, no statistical differences were found, except for hyperplastic polyps (Mann–Whitney test, *p*-value = 0.037).

Methylated NEUROG1 was comparable in non-advanced adenomas and no neoplasia which included those with no colorectal findings and benign pathologies. However, individuals bearing AA exhibited increased methylation, resulting in significant differences compared to no colorectal findings, no neoplasia, and even non-advanced adenomas (Mann–Whitney tests, *p*-values < 0.001). In a detailed analysis according to the characteristics of adenomas (Table 2), we found that individuals with adenomas sized ≥10 mm and with villous component registered elevated methylated NEUROG1 in contrast to small and tubular adenomas (Mann–Whitney tests, *p*-values < 0.001). Differences were absent between 1–2 vs. 3 or more adenomas, or distal vs. only proximal location.

Regarding CRC, increased NEUROG1 methylation was also evidenced, with statistically significant differences when compared to no colorectal findings group and no neoplasia (Mann–Whitney tests, *p*-value = 0.026 and *p*-value = 0.011, respectively). Differences were maintained when considering AN as a whole (Mann–Whitney test, *p*-value < 0.001), suggesting its utility for the detection of both CRC and AA.

### 3.3. Diagnostic Performance of Serum NEUROG1 Methylation for the Detection of Advanced Neoplasia and Advanced Adenomas

The discriminatory capacity of NEUROG1 was assessed by ROC curve analyses. The AUC for the detection of AN was 0.674 (95% CI 0.631–0.715), while for AA it resulted 0.666 (95% CI 0.622–0.707). As shown in Table 3, using >1.3518% as the NEUROG1 cut-off (90.60% specificity), 33.33% of AN and 32.08% of AA were identified, detecting 50% of CRC cases. However, the increase in specificity to 95.30% resulted in a considerable loss of sensitivity (17.54% for AN and 15.09% for AA). Proximal lesions were better detected compared to distal ones, though differences were not statistically significant for any of the cut-offs.

### 3.4. Evaluation of a Diagnostic Model Including Serum NEUROG1 Methylation and FIT

The diagnostic performance of NEUROG1 in combination with FIT was tested since fecal hemoglobin concentration results in the screening population were available. Older and male patients showed higher fecal hemoglobin concentrations, which were statistically significant. No differences regarding familial risk were found.

To account for the impact of age and gender, the linear predictors of the logistic regression models using age, gender and each of the markers as regressors were used. Based on this, the resulting AUC for the model including FIT, age and gender was 0.742 (95% CI 0.702–0.780) for AN, and 0.724 (95% CI 0.682–0.762) for AA. The inclusion of NEUROG1 methylation in the model increased the AUC up to 0.810 (95% CI 0.773–0.843) for the discrimination of AN, and 0.796 (95% CI 0.758–0.830) for AA. When AUCs from the FIT model and the NEUROG1 + FIT model were compared, statistically significant differences were found for both AN and AA (DeLong, *p*-values = 0.006). The diagnostic performance of the models is summarized in Table 4.

To assess the contribution of the variables age and gender in the models, we elaborated a new model including only these variables. The AUC resulted 0.674 (0.608–0.741) and 0.684 (0.618–0.749) for the detection of AN and AA, respectively. Additionally, age + gender models were compared to NEUROG1 + FIT (+ age + gender), resulting in statistically significant differences for AN and AA (likelihood ratio tests, *p*-values < 0.0001). These results indicate that the latter model fits significantly better than the age + gender models.

Furthermore, the superior performance of NEUROG1 + FIT was evident especially for the fixed 98.21% specificity. At this value, FIT model detected 29.82% of AN, including 75% of CRC cases, while the model including NEUROG1 resulted positive in 40.35% of AN, with all CRC cases detected (McNemar test, *p*-value = 0.031). Regarding AA, though sensitivities at 98.21% specificity also differed between models (FIT: 26.42%; NEUROG1 + FIT: 35.85%), statistical significance was not reached (McNemar test, *p*-value = 0.063). In general, the detection of distal lesions compared to proximal was superior for the two cut-offs analyzed in both models. However, a significant difference was only found for AA in the FIT model at 98.21% specificity (distal: 35.9% vs. proximal: 0%; Fisher′s test *p*-value = 0.011).

## 4. Discussion

The detection of methylated DNA in liquid biopsies represents one of the most promising biomarkers for cancer diagnosis, and efforts have also centered in their use for CRC screening [21,22]. In this study we committed to determine the diagnostic capability of serum NEUROG1 methylation for the detection of CRC and AA in an asymptomatic family-risk screening cohort, based on known NEUROG1 increased methylation in CRC [19].

In a first approach, the CpG island in the promoter of NEUROG1 targeted in previous studies [13,19,23] was assessed by bisulfite pyrosequencing in a case-control cohort. These results allowed the selection of CpG sites 7, 8, 9 and 12, that better discriminated individuals with AN (CRC or AA) from individuals with no colorectal findings. Hence, we designed a custom nested qPCR assay for quantifying methylation at these selected sites and extended the study to an asymptomatic family-risk screening cohort.

We found that individuals with no colorectal findings and benign pathologies exhibited low serum NEUROG1 methylation. This similarity is of great value since biomarkers are frequently altered in benign pathologies, limiting their clinical utility [24]. However, individuals bearing hyperplastic polyps showed slightly increased methylation that could probably be related to the serrated carcinoma pathway [25]. This increase in NEUROG1 methylation from normal colonic mucosa to hyperplastic polyps, also progressing in adenomas, primary adenocarcinomas and metastatic adenocarcinomas, was previously evidenced in tissue [16].

In line with this, we describe for the first time a trend towards increased serum methylated NEUROG1 for the most severe characteristic of adenomas: Number (≥3), size (≥10 mm) and histology (villous component), consistent with the significant elevation of methylation in AA but not in non-advanced adenomas. As regards CRC, increased methylation was also registered, coinciding with previous results [19].

In terms of discriminatory capacity, NEUROG1 showed an AUC of 0.674 for separating individuals with AN from the rest of the cohort (no colorectal findings, benign pathologies and non-advanced adenomas), and 0.666 for AA. Sensitivity for AN ranged from 17.54 to 52.63%, detecting 50 or 75% of cancer cases, with specificity between 95.30% and 80.09%. Regarding AA, 15.09–50.94% of these lesions were detected. It should be noted that for all the cut-offs evaluated, the detection of distal and only proximal AA was comparable, with no statistically significant differences.

Many studies have examined blood-based DNA methylation markers for the diagnosis of CRC. Although methylated markers such as RASSF1A, SDC2, BCAT1, IKZF1, ALX4, SDC2 and WIF-1, among others, have demonstrated some usefulness for the detection of established cancers [17,21,22], the goal of detecting precancerous adenomas has not been achieved yet. The most known methylation marker in blood is SEPT9, which is FDA approved. The PRESEPT study [26], conducted in a real screening scenario, indicated 48.2% sensitivity for CRC with 91.5% specificity. The sensitivity of the test was reported to be increased, especially among Asians [27]. However, sensitivity for AA was very low (11.2%), slightly higher than the false positive rate for all non-cancer individuals [26], indicating no utility in detecting precancerous lesions [28].

In our study, we also analyzed the performance of NEUROG1 combined with FIT. The variations in NEUROG1 methylation found intrinsic to age and gender, together with the epidemiological fact that both AA and CRC have a higher prevalence in males and in older-aged groups, well justify the need of including these confounders in the diagnostic models.

NEUROG1 + FIT model detected all CRC cases, besides 35.85–47.17% of AA, when specificity was fixed around 98% and 95%. The diagnostic competence of this model resulted more evident when compared to the FIT model (including FIT, age and gender), proposed in other works for CRC detection in symptomatic [29] and asymptomatic [30] patients. For the FIT model, sensitivity for AN and AA considerably decreased, more abruptly for 98.21% specificity. At this cut-off, 75% of CRC cases and 26.42% of AA were detected, instead of all cancers and 35.85% of AA as seen for NEUROG1 + FIT. Therefore, the combination of methylated NEUROG1 and FIT, both non-invasive tests, could be very helpful for the detection of cancers and premalignant AA, to be incorporated into CRC screening programs. Additionally, the approximate cost per patient of NEUROG1 methylation is USD 11 and the cost of FIT is USD 5-23.

According to literature, studies combining blood methylation markers with FIT are scarce. Two of these are centered on SEPT9. Johnson and colleagues [31] reported 88.7% sensitivity for CRC and 18.5% for AA, with specificity lower than 80%. A higher sensitivity for CRC and AA (94.2% and 42.9%, respectively) was reached in another study combining methylated SEPT9 and the stool test, with 80.8% specificity [32]. On the other hand, methylated BCAT1 and IKZF1 combined with FIT showed 82% sensitivity for CRC and 25% for AA, at 73% specificity [33]. These studies do not meet the high specificity recommended for a screening test, while in our work specificity was intentionally conditioned for screening. However, to make an equitable comparison, our NEUROG1 + FIT model at 80% specificity rendered 100% sensitivity for CRC and 67.92% for AA, noticeably superior to the above studies.

Unlike other studies analyzing methylation markers using qPCR but reporting qualitative interpretations [26,34], we report relative quantifications using a custom nested MS-qPCR. This enabled us to evaluate the diagnostic performance of NEUROG1, as well as the combination with FIT, fixing specificity at the desirable level for screening. The large, asymptomatic screening cohort analyzed constitutes another strength of our study, allowing the estimation of the diagnostic capability of methylated NEUROG1 in a real-life screening scenario that included a variety of colorectal pathologies.

One of the limitations of the study is the reduced number of CRC cases, though the 0.8% prevalence corresponds to that observed in other comparable screening cohorts [35,36,37]. A larger number of asymptomatic CRC cases from screening would be desirable not only to confirm the diagnostic capacity, but also to evaluate the performance for detecting distal and proximal tumors. Additionally, among the 54 AA cases, only 14 patients had proximal lesions, limiting the analysis performed regarding the detection of distal vs. proximal lesions. On the other hand, the evaluation of NEUROG1 in other non-colorectal tumors and benign gastrointestinal pathologies would also be of utility to estimate the specificity of the biomarker.

Liquid biopsies are readily available and contain stable methylated DNA targets. In our study, we demonstrate that serum NEUROG1 methylation could be a useful test for the detection of premalignant advanced adenomas and cancer, for the screening of CRC in asymptomatic individuals. The convenient diagnostic capability of methylated NEUROG1 combined with the also non-invasive FIT results in an improved performance and should be further examined in larger studies, including average-risk individuals, to confirm the utility of the marker combination.

## 5. Patents

Some of the results reported in this paper are part of the Spanish patent ES2683866.

## Figures and Tables

**Table 1:** Serum NEUROG1 methylation according to colorectal findings in the screening cohort.

Colorectal Findings	*n*	Mean ± SD	Median (IQR)	*p*-Value ^1^	
				a	b
No neoplasia	330	1.96 ± 10.39	0.00 (0.00–0.00)		
No colorectal findings	171	2.32 ± 9.53	0.00 (0.00–0.00)		
Benign pathologies	159	1.58 ± 11.26	0.00 (0.00–0.00)	0.083	
Inflammatory polyps	4	0.00	0.00	0.229	
Hyperplastic polyps	38	3.03 ± 16.31	0.00 (0.00–0.00)	0.037	
Hemorrhoids	65	0.45 ± 1.89	0.00 (0.00–0.00)	0.122	
Diverticula	46	2.33 ± 14.73	0.00 (0.00–0.00)	0.526	
Other benign pathologies	6	0.00	0.00	0.141	
Non-advanced adenomas	117	1.15 ± 9.30	0.00 (0.00–0.00)	0.192	0.547
Advanced neoplasia	57	10.92 ± 27.79	0.00 (0.00–3.52)	**<0.001**	**<0.001**
Advanced adenomas	53	10.85 ± 28.51	0.00 (0.00–2.74)	**<0.001**	**<0.001**
Cancer	4	11.91 ± 17.93	4.87 (0.00–30.86)	**0.026**	**0.011**

SD: standard deviation; IQR: interquartile range. ^1^ *p*-value for Mann–Whitney test for comparison with no colorectal findings group (a) and no neoplasia group (b).

**Table 2:** Serum NEUROG1 methylation according to the characteristics of adenomas in the screening cohort.

Characteristic	*n*	Mean ± SD	Median (IQR)	*p*-Value ^1^
Number				
1–2	138	3.55 ± 16.40	0.00 (0.00–0.00)	0.429
≥3	32	6.84 ± 24.51	0.00 (0.00–0.32)	
Size				
<10 mm	123	2.69 ± 14.74	0.00 (0.00–0.00)	**0.001**
≥10 mm	47	8.05 ± 24.78	0.00 (0.00–2.05	
Histology				
Tubular	143	2.65 ± 14.60	0.00 (0.00–0.00)	**<0.001**
Villous component	27	12.21 ± 30.00	0.46 (0.00–6.50)	
Adenomas				
Non-advanced	117	1.15 ± 9.30	0.00 (0.00–0.00)	**<0.001**
Advanced	53	10.85 ± 28.51	0.00 (0.00–2.74)	
Location				
Distal	129	5.32 ± 20.70	0.00 (0.00–0.13)	0.680
Only proximal	41	0.56 ± 2.06	0.00 (0.00–0.00)	

SD: standard deviation; IQR: interquartile range. ^1^ *p*-value for Mann–Whitney test.

**Table 3:** Diagnostic performance of serum NEUROG1 methylation for the detection of advanced neoplasia and advanced adenomas in the screening cohort.

		Advanced Neoplasia			Advanced Adenomas			
*NEUROG1* Cut-Off (NMP)	Specificity % (95% CI)	Sensitivity % (95% CI)	NPV % (95% CI)	PPV % (95% CI)	Sensitivity % (95% CI)	NPV % (95% CI)	PPV % (95% CI)	Detection % Distal/Proximal^1^
>1.3518%	90.60 (87.5–93.1)	33.33 (21.4–47.1)	91.4 (88.4–93.9)	31.1 (19.9–44.3)	32.08 (19.9–46.3)	91.8 (88.9–94.2)	28.8 (17.8–42.1)	28.21/42.86 (NS)
>7.4194%	95.30 (92.9–97.1)	17.54 (8.7–29.9)	90.1 (87.0–92.6)	32.3 (16.7–51.4)	15.09 (6.7–27.6)	90.4 (87.4–92.9)	27.6 (12.7–47.2)	17.95/21.43 (NS)

NMP: normalized methylation percentage; NS: not significant differences for Fischer′s exact test for detection of distal vs. proximal AA. ^1^ Detection % of distal and only proximal AA.

**Table 4:** Diagnostic performance of the models for the detection of advanced neoplasia and advanced adenomas in the screening cohort.

		Advanced Neoplasia			Advanced Adenomas			
Cut-Off	Specificity % (95% CI)	Sensitivity % (95% CI)	NPV % (95% CI)	PPV % (95% CI)	Sensitivity % (95%CI)	NPV % (95% CI)	PPV % (95% CI)	Detection % Distal/Proximal
FIT >0.2684	95.75 (93.4–97.4)	45.61 ^a^ (32.4–59.3)	93.2 (90.6–95.4)	57.8 (42.2–72.3)	41.51 ^c^ (28.1–55.9)	93.243 (90.6–95.4)	53.7 (37.4–69.3)	48.72/21.43 (NS)
FIT >0.4190	98.21 (96.5–99.2)	29.82 ^b^ (18.4–43.4)	91.6 (88.8–94.0)	68.0 (46.5–85.1)	26.42 ^d^ (15.3–40.3)	91.8 (89.0–94.1)	63.6 (40.7–82.8)	35.90/0.00 *
NEUROG1 + FIT >0.2899	95.75 (93.4–97.4)	50.88 ^a^ (37.3–64.4)	93.9 (91.2–95.9)	60.4 (45.3–74.2)	47.17 ^c^ (33.3–61.4)	93.9 (91.2–95.9)	56.8 (41.0–71.7)	53.85/28.57 (NS)
NEUROG1 + FIT >0.4598	98.21 (96.5–99.2)	40.35 ^b^ (27.6–54.2)	92.8 (90.1–95.0)	72.4 (55.4–88.1)	35.85 ^d^ (23.1–50.2)	92.8 (90.1–95.0)	70.4 (49.8–86.2)	43.59/14.29 (NS)

NS: not significant differences for Fischer′s exact test for comparison of detection of distal vs. proximal AA. McNemar test for comparison of proportions based on the NEUROG1 + FIT vs. FIT: AN cases (^a^ *p*-value = 0.375; ^b^ *p*-value = 0.031); AA cases (^c^ *p*-value = 0.375; ^d^ *p*-value = 0.063). * Fisher′s test *p*-value = 0.011

## Supplementary Materials

Supplementary Materials can be found at https://www.mdpi.com/2075-4418/10/7/437/s1.

## Abbreviations

AA	Advanced Adenomas
AN	Advanced Neoplasia
AUC	Area Under the Curve
CRC	Colorectal Cancer
CIMP	CpG island methylator phenotype
Cq	Quantification Cycle
FIT	Fecal Immunochemical Test
FDR	First-Degree Relative
IQR	Interquartile Range
MS-qPCR	Methylation-Specific qPCR
NMP	Normalized Methylation Percentage
ROC	Receiver-Operating Characteristic
