# Supplementary material for: Value of Serum NEUROG1 Methylation for the Detection of Advanced Adenomas and Colorectal Cancer
Source: Diagnostics (Basel). 2020 Jun 28;10(7):437. doi: 10.3390/diagnostics10070437 (PMC7399835; doi:10.3390/diagnostics10070437)
Supplement: Supplementary file 1 [file diagnostics-10-00437-s001.zip › Supplementary material.pdf]

Supplementary material

Bisulfite pyrosequencing

PCR was performed in a 30 µL reaction mix containing 2 µL of bisulfite-modified DNA, 0.54 µM forward and reverse primers (Forward 5'-GGTAGTTAGGGTGAATGGAG-3´, Reverse 5'-/biotin/AAACCCCCTAACCAACTTAAC CC-3'), 75 µM dNTPs mixture, 2.5 mM $MgCl_2$, 1X *Epi Taq* Buffer and 1.2 unit *Takara Epi Taq* HotStart, with the following cycling conditions: 95ºC for 5 min, 37 cycles of 95ºC for 30 sec, 62ºC for 30 sec and 72ºC for 30 sec, and finally 72ºC for 7 min. All PCR products (5 µL) were run on a 3% agarose gel to verify amplification and suitable DNA amount for pyrosequencing. Single-stranded biotinylated PCR products were prepared for sequencing with the PyroMark TM Vacuum Prep Workstation (Qiagen).

A single sequencing reaction was carried out (S: 5'-GGAGAGAGGAGGTAGGTTATTTT-3') to interrogate the 12 CpG sites. In each sequencing set, besides the samples, a fully-methylated control, an unmethylated control and a no template control were included.

Methylation-specific qPCR

The pre-amplification was carried out in 25 µL containing 3 µL of bisulfite-modified DNA/controls, 0.72 µM forward and reverse primers (pyrosequencing primers without biotin), 200 µM dNTPs mixture, 2.5 mM $MgCl_2$, 1X *Epi Taq* Buffer and 1 unit *Takara Epi Taq* HotStart, as follows: 95ºC for 5 min, 24 cycles of 95ºC for 30 sec, 62ºC for 30 sec and 72ºC for 30 sec, and finally 72ºC for 7 min. In each pre-amplification set, a fully-methylated control, an unmethylated control and a no template control were included, besides the bisulfite-modified samples from the screening cohort.

In the second step, a MS-qPCR was performed to quantify methylation at the CpG sites selected by bisulfite pyrosequencing, using diluted pre-amplification products as template. The primer and probe sequences were as follows: Forward 5'-GGGAGGTYGTTATCGTTTTT-3', Reverse 5'-CGACGCCGCCGATTAA-3', Probe 5'-/6-FAM/TTAATTCGG/ZEN/AGTGTGTTCGTA ATTA/3IABkFQ/-3', corresponding to a 71 pb amplicon. MS-qPCR reactions were performed in triplicate in a 20 µL volume containing 2 µL of diluted PCR product (1:10), 0.4 µM probe,

0.6 μM forward and reverse primers and 1X *TaqMan Gene Expression Master Mix* (Applied Biosystems), as follows: 95ºC for 10 min, 40 cycles of 95ºC for 30 s, 62ºC for 30 s and 72ºC for 30 s. Reactions were carried out in a StepOne instrument (Applied Biosystems).

Normalization for input DNA across the samples with *ACTB* was also done in a nested qPCR. The pre-amplification of *ACTB* was performed in a final volume of 25 μL, as in the case of *NEUROG1*, using the following primers: Forward 5'-TCCCTTAAAAATTACAAAAAC CACA-3', Reverse 5'-TGGTGATGGAGGAGGTTTAG-3', and 60ºC as annealing temperature. The conditions for qPCR were as previous, using the same *ACTB* primers, Probe 5'-/VIC/ACCACCACCCAACACACAATAACAAACACA/MGBNFQ/-3', 1:500 dilution of the *ACTB* pre-amplification product and an annealing temperature of 60ºC (amplification efficiency: 88.89%; slope: -3.6211; R^2=0.9976).
