# Supplementary material for: Value of Serum NEUROG1 Methylation for the Detection of Advanced Adenomas and Colorectal Cancer
Source: Diagnostics (Basel). 2020 Jun 28;10(7):437. doi: 10.3390/diagnostics10070437 (PMC7399835; doi:10.3390/diagnostics10070437)
Supplement: Supplementary file 1 [file diagnostics-10-00437-s001.zip › Supplementary tables.pdf]

Supplementary Table 1. Serum NEUROG1 methylation based on bisulfite pyrosequencing in the case-control cohort.

	**No colorectal findings** (n=33)		**Advanced neoplasia** (n=48)			
**CpG site**	Mean±SD	Median (IQR)	Mean±SD	Median (IQR)	*P*-value [1]	AUC[2] (CI 95%)
**CpG 1**	2.31±5.76	0.00 (0.00-2.50)	6.53±19.15	0.00 (0.00-2.83)	0.549	0.532 (0.405-0.660)
**CpG 2**	5.47±5.89	4.55 (0.0-7.42)	11.20±22.29	4.39 (2.72-7.42)	0.839	0.513 (0.384-0.642)
**CpG 3**	2.02±3.92	0.00 (0.00-3.70)	7.39±20.83	0.00 (0.00-3.97)	0.385	0.549 (0.422-0.676)
**CpG 4**	4.12±4.06	4.09 (0.00-5.61)	9.50±21.19	4.14 (0.00-5.67)	0.718	0.523 (0.396-0.651)
**CpG 5**	1.31±3.42	0.00 (0.00-0.00)	5.07±17.92	0.00 (0.00-0.00)	0.803	0.511 (0.383-0.640)
**CpG 6**	3.01±4.62	0.00 (0.00-6.05)	9.20±21.38	0.00 (0.00-6.29)	0.615	0.529 (0.403-0.656)
**CpG 7**	4.39±4.72	4.44 (0.00-6.69)	10.10±19.64	4.73 (0.00-9.71)	0.256	**0.573** (0.448-0.699)
**CpG 8**	7.90±5.71	7.73 (4.36-12.76)	14.95±21.35	9.25 (6.06-13.39)	0.113	**0.604** (0.478-0.729)
**CpG 9**	5.78±5.97	5.82 (0.00-8.18)	12.70±20.47	7.07 (5.18-12.61)	**0.042**	**0.633** (0.511-0.755)
**CpG 10**	3.55±5.57	0.00 (0.00-5.03)	9.17±21.17	3.16 (0.00-6.47)	0.428	0.549 (0.422-0.675)
**CpG 11**	3.69±9.59	0.00 (0.00-4.79)	5.86±17.82	0.00 (0.00-2.00)	0.765	0.485 (0.355-0.614)
**CpG 12**	4.82±5.41	3.95 (0.00-8.24)	11.65±20.42	6.12 (0.00-10.57)	0.167	**0.589** (0.464-0.713)
**Mean CpG 1-12**	4.03±3.58	3.15 (1.89-4.84)	9.44±19.12	3.55 (2.51-6.82)	0.108	0.605 (0.479-0.731)
**Mean CpG 7, 8, 9 and 12**	5.72±4.43	5.69 (2.52-7.24)	12.35±19.20	6.74 (4.85-10.75)	**0.021**	**0.652** (0.531-0.772)

SD: standard deviation; IQR: interquartile range. [1]*P*-value for Mann-Whitney test for comparison of no colorectal findings and advanced neoplasia; [2]AUC for the detection of advanced neoplasia.

Supplementary Table 2. Serum NEUROG1 methylation according to demographic characteristics of the screening cohort.

Characteristics	n	Mean±SD	Median (IQR)	*P*-value[1]
**Age (years)**				
<50	169	1.18±7.09	0.00 (0.00-0.00)	
≥50	335	3.60±15.86	0.00 (0.00-0.00009)	0.059
**Gender**				
Male	205	3.26±15.29	0.00 (0.00-0.0002)	
Female	299	2.47±12.33	0.00 (0.00-0.00)	0.084
**Familial risk**				
1 FDR ≥60 years	333	2.45±12.35	0.00 (0.00-0.00001)	
1 FPG <60 years or 2 FDR	171	3.45±15.79	0.00 (0.00-0.00006)	0.358

FDR: first-degree relative; SD: standard deviation; IQR: interquartile range. [1]*P*-value for Mann-Whitney test for comparison according to groups of age, gender and familial risk.
